# Diamond Light Source: contributions to SARS-CoV-2 biology and therapeutics

**DOI:** 10.1016/j.bbrc.2020.11.041

**Published:** 2021-01-29

**Authors:** Martin A. Walsh, Jonathan M. Grimes, David I. Stuart

**Affiliations:** a Diamond Light Source Ltd., Harwell Science and Innovation Campus, Didcot, OX11 0DE, UK; b Research Complex at Harwell, Harwell Science and Innovation Campus, Didcot, OX11 0FA, UK; c Division of Structural Biology, The Nuffield Department of Medicine, University of Oxford, Headington, Oxford, OX3 7BN, UK; d Instruct-ERIC, Oxford House, Parkway Court, John Smith Drive, Oxford, OX4 2JY, UK

**Keywords:** SARS-CoV-2, COVID-19, Crystallography, Structural biology, Fragment based drug discovery, Cryo-electron microscopy, Drug repurposing, Structure-based drug design, Antibody structure

## Abstract

The impact of COVID-19 on public health and the global economy has led to an unprecedented research response, with a major emphasis on the development of safe vaccines and drugs. However, effective, safe treatments typically take over a decade to develop and there are still no clinically approved therapies to treat highly pathogenic coronaviruses. Repurposing of known drugs can speed up development and this strategy, along with the use of biologicals (notably monoclonal antibody therapy) and vaccine development programmes remain the principal routes to dealing with the immediate impact of COVID-19. Nevertheless, the development of broadly-effective highly potent antivirals should be a major longer term goal. Structural biology has been applied with enormous effect, with key proteins structurally characterised only weeks after the SARS-CoV-2 sequence was released. Open-access to advanced infrastructure for structural biology techniques at synchrotrons and high-end cryo-EM and NMR centres has brought these technologies centre-stage in drug discovery. We summarise the role of Diamond Light Source in responses to the pandemic and note the impact of the immediate release of results in fuelling an open-science approach to early-stage drug discovery.

## 1 Introduction

The rapid spread of a novel coronavirus led to the second pandemic of the 21^st^ century being declared by the World Health Organisation on March 11, 2020 [1]. This virus, SARS-Cov-2 [2], has been directly responsible for over 1.2 million deaths as of November 1, 2020 [3]. Furthermore the world economy has been severely affected [4,5]. Although two previous coronavirus outbreaks causing severe disease have occurred since 2002, namely severe acute respiratory syndrome (SARS) and Middle East respiratory syndrome (MERS), no antivirals or vaccines have been approved to combat these and probable future zoonotic coronavirus outbreaks. As observed in April by Stephen Burley, this represents market failure [6]. Nevertheless the COVID-19 pandemic has generated an unprecedented response from the scientific community – currently more than 95,000 publications on SARS-CoV-2 research (https://www.who.int/emergencies/diseases/novel-coronavirus-2019/global-research-on-novel-coronavirus-2019-ncov). With the knowledge accumulated since the first SARS outbreak in 2002 this is transforming our understanding of coronaviruses, from genome organisation [7,8] to cell entry, replication, release and pathogenesis [9].

**Fig. 1:**
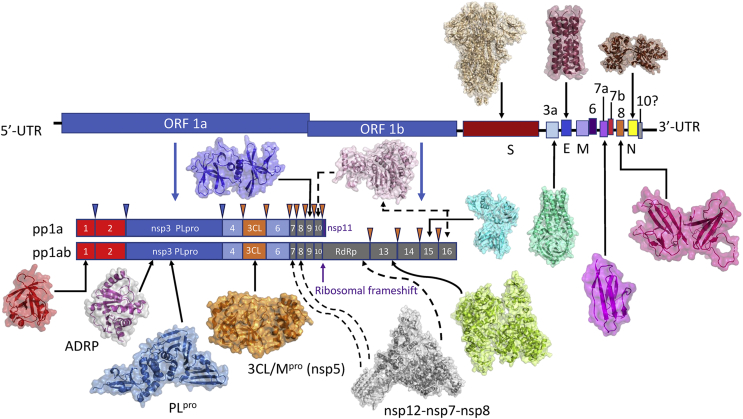
Schematic of the RNA genome of SARS-CoV-2 showing the 3D structural coverage to date. Cleavage sites indicated by orange and blue arrowheads for M^pro^ and PL^pro^, respectively. The boxes for the ORFs are approximate and the boxes representing the 6 accessory proteins are larger for clarity [8]. The following PDBs were used to generate the representative structures: 7K7P; 6WEN [65]; 6WRH; 6YB7 [14]; 6YYT [15]; 6WXD [66]; 6W4H [67]; 6ZSL; 6VWW [68]; 6VXX [69]; 7KEG [70]; 6WZQ [71]; 6XDC [72]; 6W37; 7JTL [73].

The speed of response from the structural biology community was remarkable. The deposition of the SARS-Cov-2 genome sequence [[10], [11], [12], [13]] on January 11, 2020 made it immediately available globally, and the structural biology community set to work. The groups of Zihe Rao and Haitao Yang quickly determined the first structure, which was deposited in the PDB on January 26 and officially released on February 5. Now there are over 500 depositions of 16 different proteins (as of October 28, 2020, Table 1). The structural coverage (Fig. 1), includes several attractive drug targets. We have learnt, notably from the response to HIV, that given time and dedication it is possible to devise effective therapies against difficult to tackle RNA viruses. For HIV-1 those therapies are underpinned by drugs acting against the viral polymerase and the protease, both targets are now accessible for SARS-CoV-2 [14,15]. In addition, the structure of the receptor binding glycoprotein (another successful HIV-1 target) has been determined and a potential druggable site identified [16,17].

**Table 1:** Summary of Protein structures determined from SARS-CoV-2 and collated in the Protein Data Base Europe Knowledge Base (PDDe-KB) [75].

Protein	Structures determined	PDBe-KB COVID-19 Data Portal
Nsp1	14	https://www.ebi.ac.uk/pdbe/pdbekb/protein/PRO_0000449619
Nsp2	0	https://www.ebi.ac.uk/pdbe/pdbe-kb/protein/PRO_0000449620
Nsp3	19 (PL^pro^) 12 (ADRP)^a^	https://www.ebi.ac.uk/pdbe/pdbe-kb/protein/PRO_0000449621
Nsp4	0	https://www.ebi.ac.uk/pdbe/pdbe-kb/protein/PRO_0000449622
Nsp5 (M^pro^)	197	https://www.ebi.ac.uk/pdbe/pdbe-kb/protein/PRO_0000449623
Nsp6	0	https://www.ebi.ac.uk/pdbe/pdbe-kb/protein/PRO_0000449624
Nsp7	19	https://www.ebi.ac.uk/pdbe/pdbe-kb/protein/PRO_0000449625
Nsp8	19	https://www.ebi.ac.uk/pdbe/pdbe-kb/protein/PRO_0000449626
Nsp9	4	https://www.ebi.ac.uk/pdbe/pdbe-kb/protein/PRO_0000449627
Nsp10	21	https://www.ebi.ac.uk/pdbe/pdbe-kb/protein/PRO_0000449628
Nsp12	21	https://www.ebi.ac.uk/pdbe/pdbe-kb/protein/PRO_0000449628
Nsp13	53	https://www.ebi.ac.uk/pdbe/pdbe-kb/protein/PRO_0000449630
Nsp14	0	https://www.ebi.ac.uk/pdbe/pdbe-kb/protein/PRO_0000449631
Nsp15	11	https://www.ebi.ac.uk/pdbe/pdbe-kb/protein/PRO_0000449632
Nsp16	18	https://www.ebi.ac.uk/pdbe/pdbe-kb/protein/PRO_0000449633
S	136	https://www.ebi.ac.uk/pdbe/pdbe-kb/protein/P0DTC2
E	1	https://www.ebi.ac.uk/pdbe/pdbe-kb/protein/P0DTC4
M	0	https://www.ebi.ac.uk/pdbe/pdbe-kb/protein/P0DTC5
N	13	https://www.ebi.ac.uk/pdbe/pdbe-kb/covid19/P0DTC9
ORF3a	1	https://www.ebi.ac.uk/pdbe/pdbe-kb/covid19/P0DTC3
ORF6	0	https://www.ebi.ac.uk/pdbe/pdbe-kb/protein/P0DTC6
ORF7a	1	https://www.ebi.ac.uk/pdbe/pdbe-kb/covid19/P0DTC7
ORF7b	0	https://www.ebi.ac.uk/pdbe/pdbe-kb/protein/P0DTD8
ORF8	2	https://www.ebi.ac.uk/pdbe/pdbe-kb/protein/P0DTC8
ORF10	0	https://www.ebi.ac.uk/pdbe/pdbe-kb/protein/A0A663DJA2

a Papain-like protease domain (PL^pro^) and ADP-ribose-1’’-phosphatase domain (ADRP).

The first, repurposed, drug against HIV-1, was available after four years and it took another eight years to launch an effective triple therapy, including a protease inhibitor, a success story for structure-based drug discovery [18]. The response to SARS-CoV-2 has been far quicker. Clearly one reason is that we were ‘primed’ by prior coronavirus outbreaks, SARS-CoV-1 and MERS, so that tricks for protein production could be immediately adopted (eg [19]). But underpinning this acceleration are massive technological changes in structural biology, the democratisation of access to sophisticated tools and the unprecedented mobilisation of the community.

Relentless step-wise advances in technology have dramatically increased the throughput of macromolecular crystallography (MX) experiments. These include significantly improved and faster X-ray detectors (1 Hz at the time of the SARS-CoV-1 outbreak vs > 500 Hz today), fully automated/unattended data collection and automated software for structure solution [20]. This qualitative change opens up the possibility of far more effective structure-based drug design and, in particular fragment-based drug design. XChem implements this at Diamond (created by Frank von Delft and his group in collaboration with the Nuffield Department of Medicine, Oxford [21]). XChem can routinely collect and analyse 700 datasets a day on beamline I04-1 and this transformation of scale supported the development of innovative methods to detect binding events [22,23]. Alongside these step changes in crystallography a resolution revolution has made Cryo-EM a major player in the determination of macromolecular structures routinely in the range of 3–4 Å [24], and continued developments mean that genuine atomic resolution is now achievable [25,26]. Here, we highlight the central role of technical advances and large scale infrastructure in enabling the remarkable response to the pandemic, focussing primarily on the role played by Diamond Light Source.

## 2 SARS-CoV-2 main protease (M^pro^)- a prime target for drug discovery

The main viral protease (M^pro^) of SARS-CoV-2 plays an essential role in viral replication and has been extensively studied to identify inhibitors of SARS-CoV-1, MERS and now SARS-CoV-2 replication [[27], [28], [29], [30], [32], [33]]. Since it is present in all coronavirus genera it has the potential to act as a target for the development of broad-spectrum antivirals [31], so an extremely potent drug (sub nM) may well also protect against the next coronavirus incursion. Being a medium sized (33.8 kDa), stable enzyme that expresses well and crystallises readily, M^pro^ is well suited as a target for structure-based drug discovery [34,35]. M^pro^ is encoded by non-structural protein 5 (nsp5) and is responsible for the proteolytic cleavage at 11 sites of the polyproteins pp1a and pp1ab (Fig. 1) [36].

**Fig. 2:**
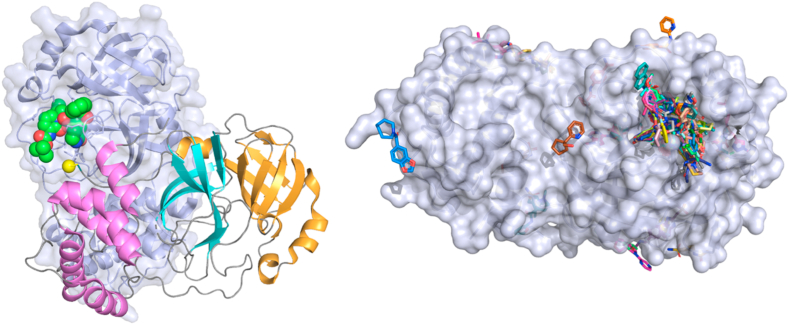
a Cartoon of the M^pro^ dimer using the 1.25 Å resolution ligand free structure PDB 6YB7 determined at Diamond. For one monomer the secondary structure features for domains I, II, and III, are shown in orange, cyan, and violet, respectively. The active site of the rear monomer is indicated by the presence of a peptide-based inhibitor, superimposed from PDB 6Y2F [74]. A yellow sphere indicates Ser1 that completes the active site. **b** Surface of the M^pro^ monomer superposed with 115 fragment hits identified from fragment screening [14].

Capitalising on their extensive knowledge of the structural biology of SARS nsps and in particular M^pro^, Zihe Rao and Haitao Yang mobilised their groups in Shanghai to target the structure of M^pro^ and other key SARS-CoV-2 replicase proteins. Remarkably they were able to determine the structure of M^pro^ in complex with a covalent inhibitor N3 [37] by mid-January 2020, using beamline BL17U1 [38] at the Shanghai synchrotron facility. In parallel they had initiated virtual and high-throughput drug screening of more than 10,000 compounds - including approved drugs and compounds in clinical trials - as inhibitors of M^pro^. As the Shanghai Synchrotron was going into a scheduled shutdown, on the 25^th^ January, the investigators contacted us to see if Diamond facilities could assist and, in particular, whether an X-ray fragment based drug discovery campaign could be initiated using the XChem platform. In the first instance the aim was to complement the efforts in Shanghai at fast tracking repurposing of drugs while bringing us on board to lay the groundwork for developing, *ab initio,* antivirals targeting SARS-CoV-2 M^pro^. Unfortunately, the lock-down in China prevented the rapid exchange of materials, and we were forced to re-clone and overexpress using their protocols [34]. By mid-February we had obtained well diffracting, robust, ligand-free crystals of M^pro^ suitable for soaking in low molecular weight fragments to carry out a high-throughput fragment screening XChem campaign [39], (Fig. 2a). A large-scale screen of electrophile (a collaboration with Nir London, Weizmann Institute, Israel) and non-covalent fragments through a combined mass-spectrometry and X-ray approach resulted in 71 hits that comprehensively sampled the active site of M^pro^, and 3 hits at the dimer interface [14]. This wealth of information enabled us to rapidly elaborate these initial weakly binding hits into more potent inhibitors through merging covalent and non-covalent fragments (Fig. 2b). To maximise the impact of the results, we released the data immediately, through a dedicated portal (https://www.diamond.ac.uk/covid-19.html) and deposition of the data with the PDB. The open release of the data was publicised through twitter on March 7^th^, leading to the establishment of a novel crowdsourcing, non-profit, open-science drug-discovery initiative – COVID-Moonshot (https://covid.postera.ai/covid) [40]. This quickly became a large-scale, international effort comprising groups from academia and industry with the aim of building on the fragment screen results to rapidly develop potent M^pro^ inhibitors. Twitter kick-started engagement with medicinal chemists across the globe, pointing them to the experimental data and a web-based interface to easily submit drug designs. Approximately 14,000 unique compound designs from 413 contributors have now been received, nearly 1200 have been synthesised and assayed, and over 200 M^pro^ ligand structures determined. Lead compounds with IC_50_ values of less than 200 nM are currently being screened in cellular antiviral assays [41]. This work represents a novel response to the drug discovery problem, however the next stages will be crucial, there is an urgent need to increase potency by two orders of magnitude and routes for development need to be established. This approach will not provide a ‘quick fix’ but might provide antivirals effective against this and future coronavirus zoonoses.

Repurposing approved drugs certainly can provide a fast-track to a therapy. Screening of approved drugs and those with pre-clinical safety data has been facilitated by the ReFrame library [42,43] of 12,000 compounds approved for clinical investigation in humans, including all currently approved drugs. We have collaborated with Exscientia (https://www.exscientia.ai/) to test this library against several targets. A number of approved drugs have been shown to inhibit SARS-CoV-2 M^pro^ at low micromolar concentrations and show antiviral activity in cellular assays [37,44,45]. To date the most effective repurposed drug against COVID-19 is the anti-inflammatory dexamethasone [46]. The ribonucleotide remdesivir has been proposed as a potential anti-viral treatment for COVID-19, but was recently shown to have no appreciable efficacy in the WHO Solidarity study [47]. Thus, there is a clear need to discover far more potent and effective anti-virals to treat COVID-19.

## 3 Polymerase

Key to the replication of the SARS-CoV-2 is the RNA dependent RNA polymerase complex, which both copies the viral genome and also makes subgenomic negative sense RNAs that act as templates for viral messenger RNAs to express structural proteins which are the building blocks for new virus particles. These steps remain poorly understood but occur in the membrane-associated replication-transcription complex (RTC), formed by nsps 1–16 (Fig. 1). Nsp12 is the RNA-dependent RNA polymerase component and requires nsp7 and nsp8 for processivity and modulation of activity. Nsp12 adopts the canonical right-handed RNA polymerase fold, linked to an N-terminal domain implicated in nucleotidyltransferase activity [48]. One nsp7 and two nsp8 molecules form a complex with each nsp12. A number of publications have revealed the overall architecture of the SARS-CoV-2 nsp12/7/8 complex, and the structural changes that occur on RNA binding [15,[49], [50], [51], [52], [53]].

**Fig. 3:**
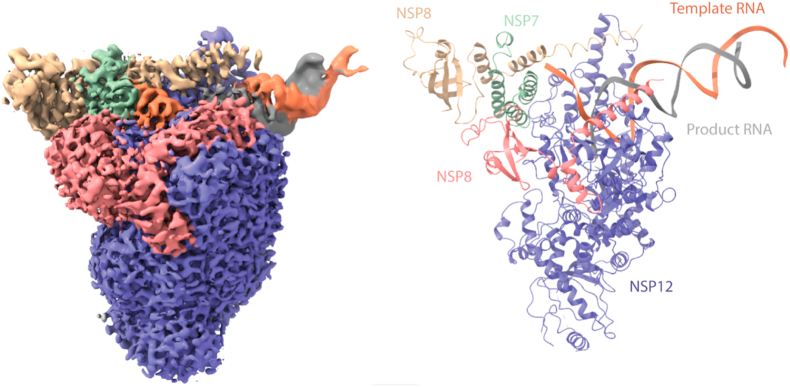
Single particle analysis cryo-EM map of nsp12/7/8 complex at a resolution of 2.8 Å (left panel) with map coloured by molecular component defined in the cartoon representation (right panel).

Early in February 2020 we ordered the synthetic genes for nsp12, nsp7 and 8, and by the 9^th^ of March were able to visualise protein bands on a gel. Nsp7 and nsp8 were expressed in bacteria and nsp12 in insect cells, with the complex formed by mixing nsp7, nsp8 and nsp12 in a molar ratio of 2:2:1. Initially cryoEM analyses were hampered by the tendency of the complex to fall apart during grid preparation. We overcame this problem by addition of an overhanging RNA duplex of sufficient length (34/40mer) – recapitulating the work by the group of Patrick Cramer [15] (Fig. 3). Work continues to understand if and how the polymerase forms higher order complexes with other nsps that are thought to be involved in processing the viral RNA in both replication and cap synthesis for mRNA synthesis. These include nsp10 which is a co-factor for nsp14 (N7-methyltransferase/3′–5′ exonuclease activity) and for nsp16 (2′-O-methyltransferase) as well as nsp13 (helicase) and nsp15 (Endoribonuclease/5′ triphosphatase). Alongside the structural work we have developed an *in vitro* RNA synthesis assay which we are using to screen for polymerase inhibitors [54].

## 4 Spike

**Fig. 4:**
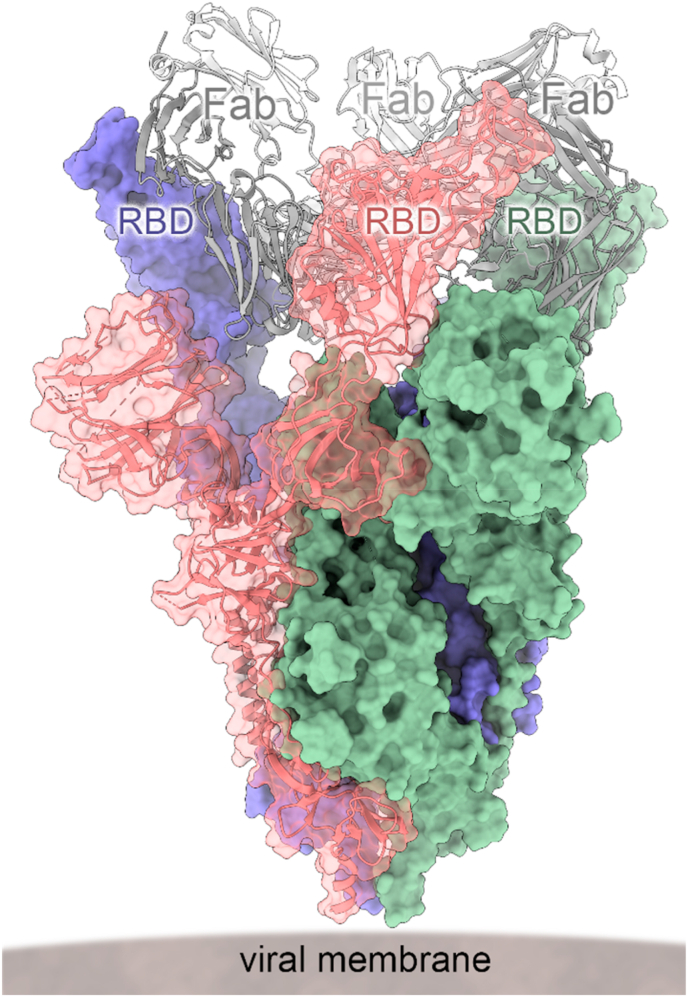
Cryo-EM structure of the SARS-CoV-2 spike in complex with Fabs of EY6A, identified by Arthur Huang. Spike trimer chains are coloured separately and shown in surface representation, one subunit semi-transparent. The Fabs are shown as grey cartoons [57]. In this complex all RBDs are locked in the up conformation by interactions with the Fabs.

The coronavirus spike (See in Table 1) is trimeric with each subunit comprising some 1200 residues. It attaches to the host receptor and is cleaved into two chains (S1 and S2) to form a metastable structure which undergoes massive rearrangements to fuse the host and virus membranes, releasing the viral genome into the host cell. It is challenging to express a soluble form of the protein that remains trimeric and does not convert into the post-fusion state. Prior studies of homologues allowed these problems to be rapidly overcome and the McLellan laboratory reported a structure for the prefusion form in March [19]. Our work on this system began in February when it became clear that there was immediate value in understanding how human antibodies interact with the spike, to inform the selection of potential therapeutic antibodies. In addition we realised that high quality spike antigen would be an invaluable reagent for the high throughput ELISA platform we were developing to test immune responses [55]. The centre of gravity for this work was the Division of Structural Biology in the Nuffield Department of Medicine, Oxford, but the collaboration included other university departments, Diamond, the Rosalind Franklin Institute, Public Health England (Porton Down), and the Chang Gung Memorial Hospital, Taiwan [[56], [57], [58]]. We have since determined some two dozen structures by both crystallography and cryo-EM, mainly of antibody complexes. This has not been entirely straightforward and interestingly, the highest resolution analyses have come from crystals of binary or ternary complexes of spike fragments (the receptor binding domain (RBD)) with Fab fragments. The cryo-EM analyses, of full-length glycosylated spike ectodomain in complex with Fabs or IgGs of monoclonal antibodies have clarified the impact of antibody binding on spike structure-function, but have often been limited by flexibility, preferred orientations, or spike destruction [56,58]. Work has meanwhile been carried out by others across the world, and a reasonably consistent picture is emerging from studies of monoclonal antibodies from convalescent and recovered patients [[59], [60], [61]]. The majority of antibodies that recognise spike do not neutralise. Most neutralising antibodies bind to the small receptor binding domain, which lies at the top of the spike, usually folded down, but capable of reaching up to engage the cell receptor, ACE2. These neutralising antibodies appear to work by preventing attachment to the ACE2 receptor. However the results of our collaborative research on antibodies CR3022 and EY6A demonstrated another route to neutralisation, through destabilisation of the prefusion spike [56,58] (Fig. 4). Such observations are of interest since numerous clinical trials are planned or underway to investigate treatment with monoclonal antibodies (usually in a cocktail). An understanding of how antibodies bind, whether they compete, and how likely it is that the epitope might mutate to escape neutralisation, is useful in choosing an effective combination. The neutralising non-ACE2 blocking epitope is concealed when the receptor binding domain is packed down, being involved in protein-protein interactions within the spike, perhaps reducing the likelihood of escape mutations being viable [58]. There is also considerable interest in the potential of nanobodies, camelid antibody fragments about 1/12th the size of an IgG molecule. These may have direct therapeutic application, and their small size might allow them to be administered intranasally [62]. Work centered at the Rosalind Franklin Institute engineered nM binders through affinity maturation [56] and ongoing work aims to produce more potent molecules.

## 5 Discussion and outlook

The COVID-19 pandemic exemplifies science as a model for global cooperation. Structural biology infrastructure can strengthen communities [63] and international organisations (such as Instruct-ERIC and EMBL in Europe) play a role, as does EU funded trans-national access (e.g. iNEXT-Discovery). To provide a single exemplar, Instruct was able to respond rapidly to the COVID-19 pandemic (see https://instruct-eric.eu/covid19) and, at the request of UK government funders and Wellcome, Instruct repurposed its ARIA access management system to provide a web platform to offer SARS-CoV-2 reagents, sourced from UK research institutions, to research groups mainly in the UK and Europe. This accelerated research by providing quality-controlled reagents and reducing duplication of effort (https://covid19proteinportal.org/).

The major structural biology infrastructures have been largely able to remain functional during lockdowns, facilitated by automation and remote access. Several special, rapid access calls were launched. In Diamond all beamlines and microscopes were available for COVID-19 research, and this remains a priority, 60 applications have been awarded time, and frequently applications have been granted multiple experimental sessions, sometimes spanning multiple techniques. Projects have used a range of instruments, including VMXi, I03, I04, I04-1, I24, B21 and B24, but principally either cryo-EM (with 174 days of microscope time used), or crystallography. Notably ∼13,000 XChem data sets have been collected, covering in addition to that described above, eight further fragment campaigns. For the future there is tremendous scope for correlative imaging and cryo-tomography. A glimpse of the prospects comes from Peijun Zhang’s group, who have imaged the SARS-CoV-2 infection process at the cellular, sub-cellular and molecular level using a combination of soft X-ray tomography, cryoFIB/SEM, cryo-electron tomography and sub-tomogram averaging using correlative workflows. These methods are a major focus for Diamond and we aim to accelerate development by co-location [64].

In summary, the rapidity with which relevant structures were determined demonstrates that structural biology is fit for purpose as a central component of the response to emerging/re-emerging viruses. Much will be done to hone the methods, but the larger challenge is to join up all the dots beyond structural biology, avoid market failure and produce effective drugs far more rapidly than has been possible in the past. Some of these challenges are discussed in [6]. We argue that there is an urgent need to establish partnerships to identify and remove bottlenecks and de-risk the process, from surveillance through to virology, biochemistry and structural studies back to chemistry, virology and regulation. This is a moral imperative, and in these difficult times it was good to hear the UK Prime minister recognising the divisions caused by COVID-19 in an address to the United Nations on September 26: ‘here in the UK … we are determined to do everything in our power to work with our friends across the UN, to heal those divisions and to heal the world’. We expect that science will continue to lead this process by example.

## Funding

This work was supported by the Oxford COVID-19 donation fund, repurposed funds from 10.13039/100014013UKRI and 10.13039/100004440Wellcome, the 10.13039/501100005150Chinese Academy of Medical Sciences and the Schmidt Foundation.

## Declaration of competing interest

We declare no competing interests.
